# Myeloperoxidase regulates hypoxia-induced inflammation and oxidative stress in liver-spleen axis

**DOI:** 10.1186/s12950-026-00503-y

**Published:** 2026-05-02

**Authors:** Yonggui Li, Xiaoli Liu, Lingming Zhang, Xiaoluan Li, Zeyi Zuo, Qiao Gao, Qinfang Zhu

**Affiliations:** 1 Department of Endocrinology, Qinghai Provincial People’s Hospital, Xining, 810007 China; 2 Clinical Medical College, Qinghai University, Xining, 810001 China

**Keywords:** Hypoxia, Myeloperoxidase, Liver-spleen axis, Oxidative stress, Inflammation

## Abstract

**Background:**

High-altitude hypobaric hypoxia induces inflammation and oxidative stress, yet the role of myeloperoxidase (MPO) in this pathology remains incompletely understood. This study aimed to investigate whether MPO mediates injury to the liverspleen axis under hypoxic conditions.

**Results:**

Using a 3day murine hypoxia model, we unexpectedly found that MPO deficiency exacerbated, rather than mitigated, damage to the liverspleen axis. Compared with hypoxic wildtype mice, MPO^−/−^ mice displayed aggravated histopathological injury, accompanied by excessive phagocyte recruitment and elevated expression of key chemokines (KC, MCP1, MIP2) and proinflammatory mediators (TNFα, IL1β, IL17A). At the molecular level, MPO absence increased splenic protein expression of NFκB, NLRP3, and iNOS, while dysregulating the antioxidant response via the NRF2/HO1 pathway.

**Conclusions:**

These results reveal a novel protective role for MPO during hypoxic stress, where it functions to moderate the innate immune response and limit collateral tissue damage in the liverspleen axis. The study provides new insights into the complex immunomodulatory functions of MPO and suggests its activity is essential for maintaining immune homeostasis during acute hypoxia.

## Background

The high-altitude environment, characterized by hypobaric hypoxia, poses a significant physiological challenge to organisms [1]. Acute exposure to such conditions disrupts the redox balance, leading to elevated oxidative stress, diminished antioxidant capacity, and a surge in endogenous reactive oxygen species (ROS), which can inflict damage on lipids, proteins, and DNA [2]. Substantial evidence underscores a vicious cycle between oxidative stress and inflammation, wherein these two pathophysiological processes mutually reinforce each other [3–5]. Inflammatory cells release abundant ROS, exacerbating oxidative damage to tissues. Conversely, ROS and oxidative stress byproducts can activate pro-inflammatory signaling pathways, such as nuclear factor kappa-B (NF-κB) [6]. For instance, hypoxia-induced oxidative stress has been shown to aggravate colon injury in wild-type mice via the activation of NF-κB and the NLRP3 inflammasome [7]. Beyond localized tissue damage, high-altitude hypoxia can systemically impair immune function [8], with reports indicating heightened immune cell infiltration and inflammatory responses in multiple organs, contributing to the pathogenesis of acute mountain sickness [9, 10].

Among the organs susceptible to hypoxic insult, the liver and spleen are of particular relevance. The liver, a central metabolic and detoxification organ, is highly vulnerable to dysfunction due to its pivotal role in circulation and its diverse functionalities. Hepatic zonation and physiology are regulated by multiple signaling pathways, including oxygen-sensing systems [11]. Uniquely, the liver receives a dual blood supply—partially deoxygenated blood from the portal vein and oxygen-rich blood from the hepatic artery. Under hypoxic conditions, hepatocytes upregulate hypoxia-inducible factor (HIF)-dependent gene transcription, a mechanism critically involved in the progression of liver fibrosis and chronic liver diseases [12, 13].

The spleen, as the largest secondary lymphoid organ, is a primary site for stress erythropoiesis and is intimately involved in adaptation to hypoxic stress [14, 15]. The strategic localization of innate and adaptive immune cells within its distinct compartments enables the spleen to orchestrate systemic immune responses [14]. Intriguingly, hypoxic and immune responses are closely intertwined [15], and pro-inflammatory cytokines can directly stimulate erythropoiesis [16, 17]. Exposure to high-altitude hypoxia frequently induces splenic contraction [18, 19], which may compromise normal immune function [19]. Animal studies suggest that up to 40% of the increase in circulating red blood cells during acute hypoxia may originate from the spleen [20].

Critically, the liver is also a pivotal player in immune and inflammatory responses [21], while splenic enlargement is recognized as a key regulator of inflammation-related immunity [22]. This functional crosstalk underscores the concept of a “liver-spleen axis,” which is evidenced by the correlation between liver pathology and spleen size in non-alcoholic fatty liver disease (NAFLD) patients [23] and by its reported dysfunction in COVID-19 [24]. While most previous studies have focused on isolated organ injury, the dynamic interplay between the liver and spleen under hypoxic conditions and its key regulatory factors remains elusive.

Myeloperoxidase (MPO), a heme peroxidase highly expressed in polymorphonuclear neutrophils (PMNs), emerges as a potential key modulator at the intersection of oxidative stress and inflammation. Neutrophils generate superoxide anions (O₂^−^) via NADPH oxidase, which are subsequently converted to hydrogen peroxide (H₂O₂) [25, 26]. MPO then utilizes H₂O₂ to produce hypochlorous acid (HOCl), a potent microbicidal agent [27]. Beyond its fundamental role in innate immunity [28], MPO is increasingly recognized as a biomarker for risk stratification in cardiovascular diseases [29, 30], and functions as a bidirectional regulator of inflammation and oxidative stress [31].

Given the critical role of neutrophils in MPO-mediated tissue damage, we sought to investigate upstream regulators of neutrophil recruitment under hypoxic conditions. IL-17A, a master regulator of neutrophil mobilization and activation [32], has been implicated in hypoxic inflammation and various liver-spleen pathologies [33, 34]. Therefore, we examined IL-17A levels in the liver and spleen to assess its potential contribution to MPO-mediated inflammation in the liver-spleen axis under hypoxic stress.

In recent years, systemic physiological adaptations and the underlying molecular mechanisms of hypoxic stress have become a central focus in high-altitude medicine. Herein, we simulated a 5,000-meter plateau environment using a hypobaric chamber to establish a murine model of acute hypoxia. By employing genetically engineered mice, this study aims to elucidate the specific impact of MPO on inflammation and oxidative stress within the liver-spleen axis under hypoxic conditions.

## Materials and methods

### Mouse experiments

Female wild-type (C57BL/6) and MPO^−/−^ mice (6–8 weeks old) were obtained from Beijing Vitro-Lux Laboratory Animal Co., Ltd. All mice were housed under specific-pathogen-free conditions in an Individual Ventilated Cage System (H6, Su Hang Technology Equipment Co., Ltd.) at 22 ± 2 °C with a 12-h light/dark cycle, with free access to autoclaved food and water. After one week of acclimatization, mice were randomly assigned to four groups (*n* = 8 per group): control (CON), hypobaric hypoxia (H), MPO^-/-^ (M), and hypobaric hypoxia MPO^-/-^ (HM). Mice in the H and HM groups were placed in a hypobaric chamber (DYC-300; Guizhou Feng Lei Oxygen Chamber Co., Ltd.) to simulate a high-altitude environment of 5,000 meters (atmospheric pressure: 52.9 kPa, approximately 10.8% O₂) for 72 hours. The CO₂ concentration was maintained below 1,500 ppm. Mice in the CON and M groups were maintained under normoxic conditions. Under normoxic conditions, MPO-KO mice were visually indistinguishable from WT controls in terms of general appearance and behavior, consistent with previous reports demonstrating normal development of MPO-deficient mice [35, 36]. After 72 hours, all mice were euthanized for sample collection. All animal experiments were approved by the Institutional Animal Care and Use Committee of Qinghai Provincial People’s Hospital (Permit Number: (2025)-153–02) and performed in accordance with relevant guidelines and regulations.

### Measurement of spleen index

At the end of the experimental protocol, all mice were weighed and then euthanized. The spleen from each mouse was carefully excised, cleared of adherent connective tissue, and immediately weighed using a high-precision analytical balance. The spleen index was calculated by normalizing the spleen weight (mg) to the final body weight (g) of the corresponding mouse, as follows: Spleen Index =${{{\rm{Spleenweight}}\left({{\rm{mg}}} \right)} \over {{\rm{Bodyweight}}\left({\rm{g}} \right)}}$

## Analysis of blood samples

Mice’s blood samples were collected to analyze the white blood cell (WBC), the neutrophils (Neu), neutrophil percentage (Neu%), the red blood cell (RBC)count, and the hemoglobin (Hb)levels using a Mindray Fully Automatic Blood Cell Analyzer (BC-5000, Beijing, China).

## Histopathology

Following necropsy, spleen and liver tissues were fixed in 4% paraformaldehyde, embedded in paraffin, and sectioned at a thickness of 5 μm. The sections were then stained with hematoxylin and eosin (H&E) according to a standard protocol. Finally, the stained sections were observed and imaged under an Olympus BX53 microscope (Olympus, Tokyo, Japan) using cellSens Entry 1.14 software.

## Immunofluorescence microscopy

Liver tissue samples were collected, frozen in OCT compound (Sakura, Tissue-Tek, U.S.A.), and stored at −80 °C. Then, 5 μm sections were cut on a Leica CM1950 Freezing Microtome (Leica Biosystems). Tissue cryosections were fixed in ice-cold acetone, washed, and blocked with bovine serum albumin (Sigma-Aldrich, V900933). To analyze the location and abundance of macrophages and neutrophils, liver tissue slides were stained with FITC-labeled anti-mouse F4/80 (eBioscience Cat. #11–4801-81) and Cy5-labeled anti-mouse Ly6G (Biolegend Cat. #108410). DNA was stained and mounted using the 4′, 6-diamidino-2-phenylindole (DAPI). The images were captured by microscopy (Leica DFCDM4 B, Germany).

## RNA isolation and real-time quantitative PCR

Total RNA was isolated from spleen and liver tissues using TRIzol reagent (Invitrogen, 15,596,026). The concentration and purity of the RNA were determined by spectrophotometry. According to the manufacturer’s instructions, complementary DNA (cDNA) was synthesized from 1 µg of total RNA using the PrimeScript RT Reagent Kit (Takara, RR047A). Quantitative real-time PCR (qRT-PCR) was then performed using TB Green Premix Ex Taq II (Takara, RR820A) on a CFX 96 Real-Time System (Bio-Rad). The primer sequences used are listed in Table 1. The expression level of each target gene was normalized to the endogenous control β-actin and calculated using the 2^−ΔΔCT^ method.

**Table 1:** Information on primer sequences

Gene	Forward primer	Reverse primer
TNF-α	CCCTCACACTCAGATCATCTTCT	GCTACGACGTGGGCTACAG
IL-1β	TCCAGGATGAGGACATGAGCAC	GAACGTCACACACCAGCAGGTTA
IL-17A	CCACGTCACCCTGGACTCTC	CTCCGCATTGACACAGCG
KC	CCGAAGTCATAGCCACACTCAA	GCAGTCTGTCTTCTTTCTCCGTTAC
MCP1	AGCAGCAGGTGTCCCAAAGA	GTGCTGAAGACCTTAGGGCAGA
MIP2	GCAGTCTGTCTTCTTTCTCCGTTAC	GCGTCACACTCAAGCTCTG
β-actin	CATCCGTAAAGACCTCTATGCCAAC	ATGGAGCCACCGATCCACA

## Measurement of cytokine production

Cytokine levels in liver and spleen homogenates were measured by ELISA. IL-1β was measured by a Mouse IL-1β ELISA Kit (Elabscience, E-EL-M0037c) according to the manufacturer’s instructions. ELISA capture antibodies and biotinylated secondary antibodies for TNF-α (BD Cat. #557516; 558,415) and IL-17A (BD Cat. #555068; 555,067) were purchased from BD Bioscience. Standard curves were obtained using recombinant murine TNF-α (BD Cat. #554589) and IL-17A (eBioscience Cat. #14–8171-80) from BD Bioscience and eBioscience.

## Flow cytometry analysis

Single-cell suspensions of the spleen were prepared at 72 h after starting the experiment. Cells were surface-stained with fluorescent-conjugated anti-CD11b (BioLegend, 101,206), anti-Ly6G (BioLegend, 108,410), anti-F4/80 (BioLegend, 123,110), and anti-CD45 (eBioscience, clone 30-F11, 11–0451-82), and then analyzed by flow cytometry (Beckman Coulter, A00-1–1102).

## Measurement of MPO concentration and activity

Mouse serum was prepared, and MPO concentration was determined using a mouse MPO ELISA Kit (MM-0338M1, China). Supernatants from liver tissues were collected, and MPO activity was measured using a specific MPO activity kit (Nanjing Jiancheng Bioengineering Institute, China) according to the manufacturer’s instructions.

## Measurement of oxidative stress markers

Supernatants from liver and spleen tissues were obtained, and the protein content was determined using a BCA Assay Kit (Servicebio, Cat #G2026-1000 T). Specific kits (Nanjing Jiancheng Bioengineering Institute, China) were used for the measurement of malondialdehyde (MDA) levels and glutathione peroxidase (GSH-PX), superoxide dismutase (SOD), and catalase (CAT) activities according to the manufacturer’s instructions.

## Western blotting

Lysis solution was added to the liver tissues to extract total protein, quantified using a BCA Protein Assay Kit (Thermo Scientific, A53225). Equal amounts of total protein were separated using sodium dodecyl sulfate-polyacrylamide gel electrophoresis and transferred onto nitrocellulose membranes (Thermo Scientific, 88,518). After blocking with 5% skim milk for 1 h, the membranes were incubated with the corresponding primary antibodies anti-βactin (1:1000, ab115777), anti-HO-1 (1:2000, ab52947), anti-MPO (1:2000, ab208670), anti-NF-κB (1:1000, ab32536), anti-NF-κB p65 (1:1000, ab76302), anti-Nrf2 (1:1000, ab92946), anti-NLRP3 (1:1000, ab270449), and anti-iNOS (1:1000, ab178945) at 4 °C overnight. After washing three times, the membranes were incubated with an HRP-conjugated secondary antibody (1:10,000, ab6721). The immunoreactive results were visualized using an enhanced chemiluminescence system (Amersham Pharmacia Biotech, Stockholm, Sweden). Protein band images were obtained using an Amersham Imager 600 Gel Imaging System. Quantification was performed using ImageJ software.

## Immunohistochemistry

Unstained blank sections cut from paraffin-embedded blocks of liver and spleen tissues were deparaffinized and rehydrated with xylene and different ethanol concentrations, respectively. Microwave antigen retrieval was performed using sodium citrate antigen retrieval solution (C1032; Solarbio, China). Sections were incubated with primary antibodies, including anti-MPO (1:50, ab208670), anti-CD68 (1:100, ab283654), anti-HO-1 (1:50, ab52947), and anti-Nrf2 (1:100, ab92946). Proceed to the next steps following instructions for the 2-step plus Poly-HRP anti-rabbit IgG detection system with DAB solution (E-IR-R215; Elabscience, China). After dehydration and being mounted, sections were examined by an Olympus BX53 microscope (Olympus), and images were captured by Olympus cellSens Entry 1.14 software.

## Statistical analysis

GraphPad Prism software (version 8.0.2) was used for the statistical analyses. The results are expressed as the mean ± SEM. Statistical analyses were performed using Student’s t-test or one-way ANOVA followed by Tukey’s multiple comparison test. *p* < 0.05 was considered statistically significant.

## Results

### Hypobaric hypoxia induces hematological changes and enhances MPO expression

**Fig. 1:**
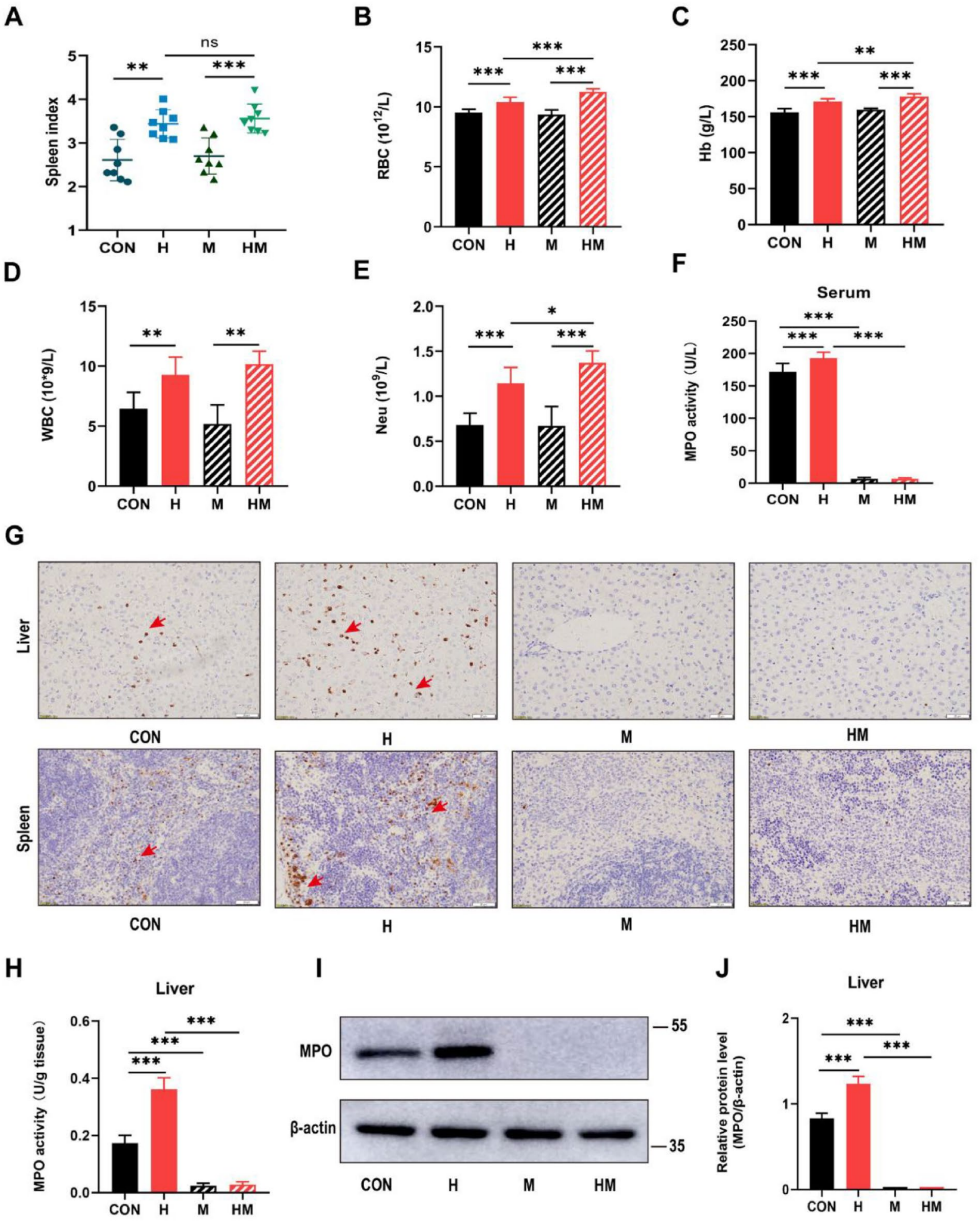
Hematological and MPO responses to hypobaric hypoxia. Mice were subjected to hypobaric hypoxia (simulating 5000 m) for 72 h. Analyses include: (**A**) Spleen index, (**B**) RBC, (**C**) Hb, (**D**) WBC, (**E**) Neu%, (**F**) Serum MPO activity (Elisa), (**G**) MPO immunohistochemistry in liver and spleen immunohistochemical analysis of MPO expression in liver and spleen (×400), (**H**) Liver MPO activity, (**I**) Representative western blot, and (**J**) Quantification of liver MPO protein. Values are mean ± SEM (*n* = 8 for A-H; *n* = 3 for J). One-way ANOVA with Tukey’s multiple comparison test was used for comparisons (*p < 0.05; **p < 0.01; ***p < 0.001)

To assess the systemic response to hypobaric hypoxia, we first examined hematological parameters and MPO expression. Hypoxia exposure for 3 days significantly increased the spleen index in both the H and HM groups compared to their respective normoxic controls (CON and M), with no significant difference observed between the H and HM groups (Fig. 1A).

Hypoxia triggered erythrocytosis, as evidenced by substantial increases in RBC count and Hb levels in both hypoxic groups (H and HM) relative to their controls (Fig. 1B, C). Notably, the HM group exhibited significantly higher RBC and Hb levels than the H group (Fig. 1B, C). Inflammatory leukocytosis was also induced by hypoxia. The WBC and the NEU percentage were significantly elevated in the H group compared to the CON group. Similarly, these parameters were higher in the HM group than in the M group (Fig. 1D, E).

Serum MPO activity was significantly upregulated in hypoxic wild-type mice (H group) but was undetectable in MPO^-/-^ mice, confirming successful genetic knockout (Fig. 1F). Immunohistochemical staining localized MPO expression primarily to the cytoplasm of phagocytic cells in the liver and spleen (Fig. 1G, arrowheads). MPO was mainly expressed in the cytoplasm of phagocytic cells [37]. Staining intensity was significantly stronger in the H group than in the CON group and was generally higher in the spleen than in the liver. Consistent with the serum results, both MPO activity and protein expression in liver tissues were markedly enhanced in the H group compared to the CON group (Fig. 1H, I, J).

### Hypoxic exposure promotes hepatic and splenic inflammation in both WT and MPO^-/-^ mice

**Fig. 2:**
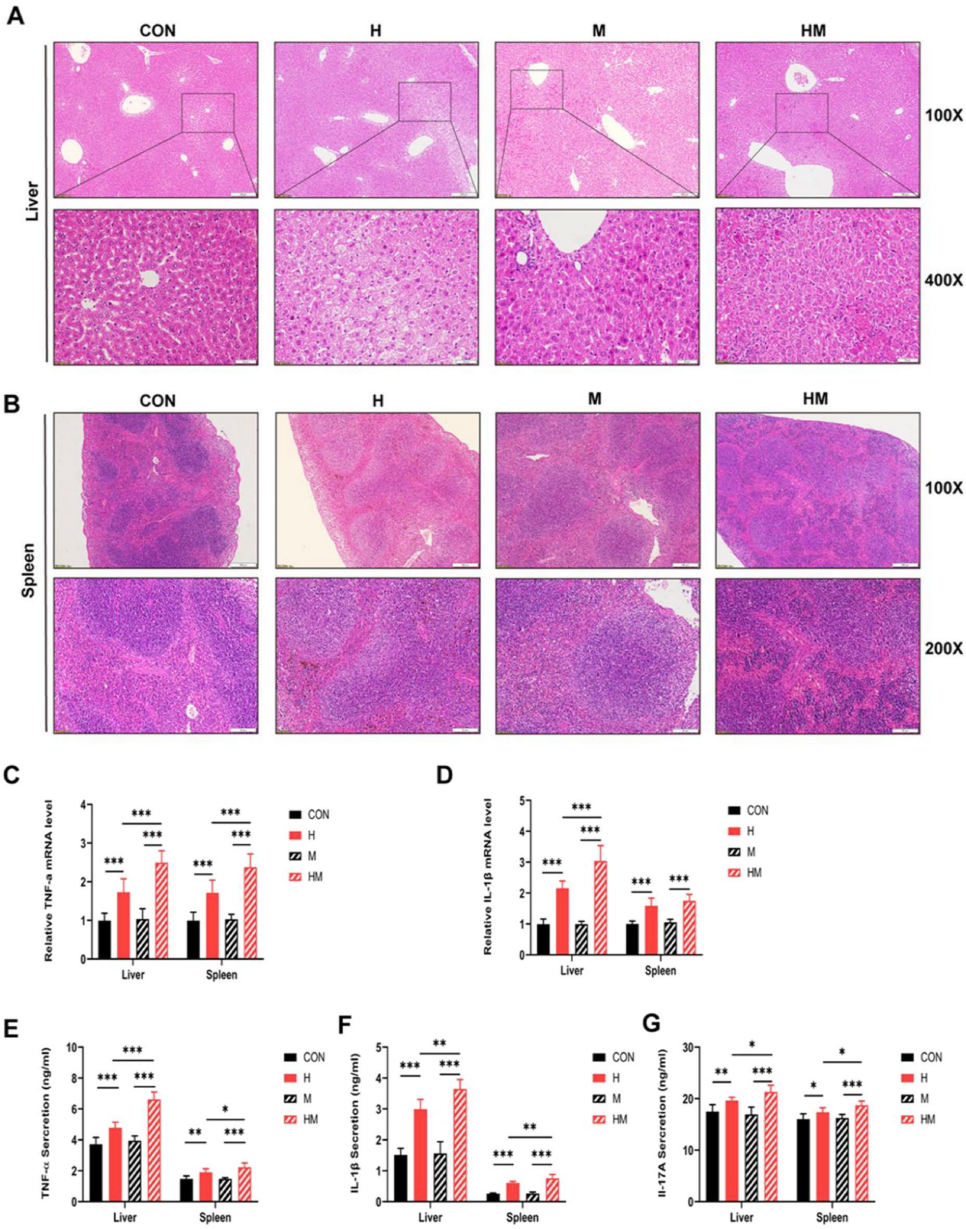
Hypoxia-induced inflammation in the liver and spleen is enhanced by MPO deficiency. (**A, B**) H&E staining of liver (**A**) (100× and 400×) and spleen (**B**) (100× and 200×) tissues. Black rectangular boxes in the 100× panels indicate the regions shown at higher magnification (400×) in the adjacent panels. qRT-PCR analysis of (**C**) TNF-α and (**D**) IL-1β mRNA expression. ELISA quantification of (**E**) TNF-α, (**F**) IL-1β, and (**G**) IL-17A protein levels. Data are presented as the mean ± SEM (*n* = 8) and analyzed via one-way ANOVA with Tukey’s multiple comparison test (*p < 0.05; **p < 0.01; ***p < 0.001)

Next, we further investigate the effect of acute hypoxia on tissue inflammation. A light microscope observed the liver and spleen’s morphology (Fig. 2A, B). Hepatic cells in the CON group were arranged in strips and radiated. The performance of the M group was similar to that of the CON group. In contrast, the H group exhibited cytoplasmic loosening in some hepatocytes and scattered chronic inflammatory cell infiltration. Notably, the HM group displayed more severe damage, characterized by disordered hepatic cords, enlarged sinusoids, and mixed inflammatory cell infiltration alongside signs of nuclear disintegration (Fig. 2A).

In the spleen (Fig. 2B), well-defined white pulp and red pulp structures were observed in the CON and M groups. Hypoxia induced hyperplasia of the white pulp and dilatation and congestion of the splenic sinuses in the H group. These pathological changes were also present in the HM group, with a more intermingled distribution of the white and red pulp.

Consistent with the histopathology, hypoxia robustly upregulated the mRNA expression of key inflammatory factors (TNF-α, IL-1β) in both the liver and spleen of WT and MPO^−^/^−^ mice (Fig. 2C, D). Importantly, the mRNA levels of TNF-α and IL-1β in the liver were significantly higher in HM mice than in H mice. In the spleen, the mRNA expression of IL-1β in the HM group showed an increasing trend compared to the H group, but this difference was not statistically significant (Fig. 2D).

This enhanced inflammatory response in the absence of MPO was further confirmed at the protein level. ELISA results showed that hypoxia significantly increased the concentrations of TNF-α, IL-1β, and IL-17A in both liver and spleen homogenates when comparing the H group to the CON group and the HM group to the M group (Fig. 2E, F, G). Crucially, the concentrations of all three cytokines were significantly elevated in the HM group compared to the H group under hypoxic conditions, indicating that MPO deficiency exacerbated the hypoxic inflammatory response.

### Hypoxia promotes phagocyte recruitment and chemokine expression in the liver, an effect potentiated by MPO deficiency

**Fig. 3:**
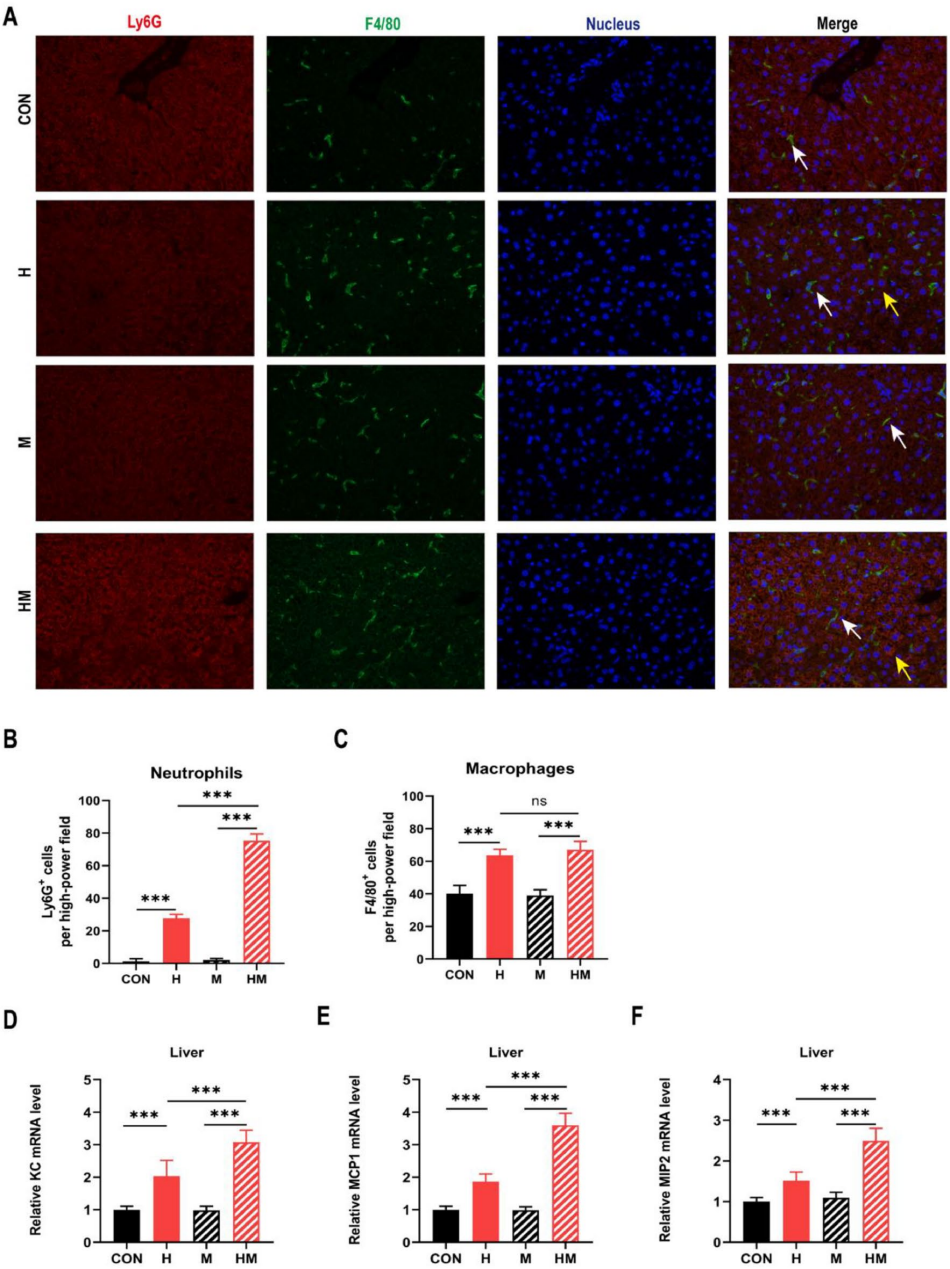
Enhanced hepatic inflammation and chemokine signaling in hypoxic MPO^-/-^ mice. (**A**) Immunofluorescence of F4/80+ (macrophages, green) and Ly6G+ (neutrophils, red) cells in liver (400×). White arrows indicate macrophages. Yellow arrows indicate neutrophils. Quantification of (**B**) F4/80^+^ and (**C**) Ly6G^+^ cells per field. qRT-PCR analysis of hepatic (**D**) KC, (**E**) MCP-1, and (**F**) MIP-2 mRNA. Data are presented as the mean ± SEM and analyzed via one-way ANOVA with Tukey’s multiple comparison test (*p < 0.05; **p < 0.01; ***p < 0.001)

To elucidate the inflammatory cell infiltration in response to hypoxia, we analyzed the recruitment of neutrophils and macrophages in the liver by immunofluorescence staining for Ly6G and F4/80, respectively (Fig. 3A, B, C). While Ly6G^+^ neutrophils were only sporadically detected in the livers of the CON and M groups, their numbers increased following hypoxic exposure. Notably, this increase was significantly more pronounced in the HM group than in the H group mice (Fig. 3A, B, C). Similarly, the population of F4/80^+^ macrophages was significantly expanded in both hypoxic groups compared to their controls, indicating a robust hypoxic inflammatory response (Fig. 3A, B, C).

To investigate the underlying mechanism for this enhanced recruitment, we measured the expression of key chemokines. Hypoxia significantly upregulated the mRNA expression of KC, MCP-1, and MIP-2 in the liver (Fig. 3D, E, F). Crucially, the expression of all three chemokines was significantly higher in the HM group than in the H group under hypoxic conditions (Fig. 3D, E, F), consistent with the observed increase in phagocyte infiltration.

### Hypoxia potentiates splenic phagocyte recruitment and chemokine expression, with an exacerbated effect in MPO-deficient mice

**Fig. 4:**
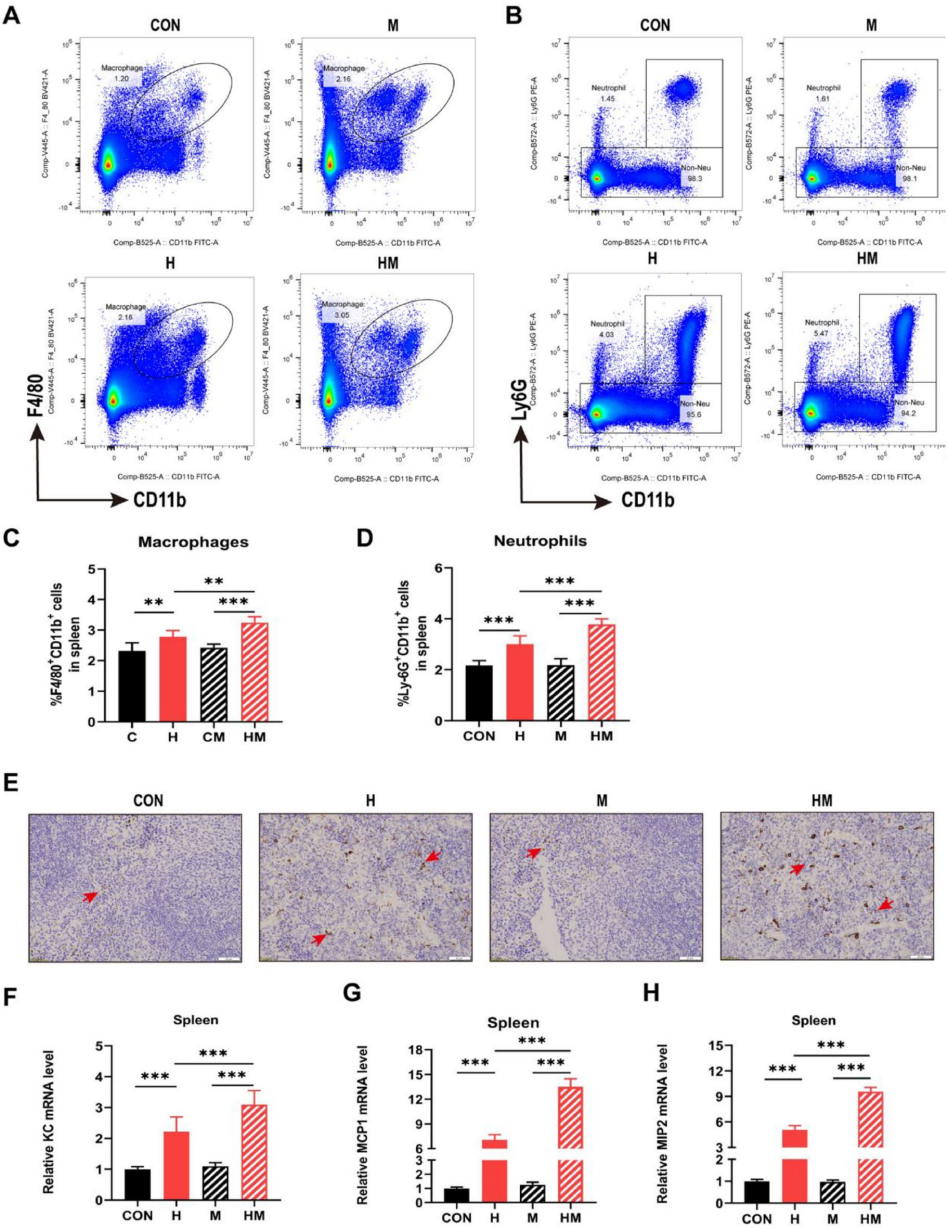
MPO deficiency exacerbates hypoxia-induced phagocyte recruitment and chemokine expression in the spleen. Splenic single-cell suspensions were analyzed by flow cytometry. Representative flow cytometry plots show the gating for (**A**) CD11b^+^ F4/80^+^ macrophages and (**B**) CD11b^+^ Ly6G^+^ neutrophils. Quantitative analysis of the percentages of (**C**) macrophages and (**D**) neutrophils. Data are presented as mean ± SEM (*n* = 6). (**E**) Representative images of immunohistochemical staining for CD68 in spleen sections (400×). mRNA expression levels of the chemokines (**F**) KC, (**G**) MCP-1, and (**H**) MIP-2 were determined by qRT-PCR (*n* = 8). Statistical significance was determined by one-way ANOVA followed by Tukey’s multiple comparison test (**p < 0.01, ***p < 0.001)

We next investigated the inflammatory response in the spleen. Flow cytometric analysis of splenic single-cell suspensions revealed that hypoxia significantly increased the proportions of CD11b^+^ F4/80^+^ macrophages and CD11b^+^ Ly6G^+^ neutrophils. Notably, this increase was significantly more pronounced in the HM group than in the H group (Fig. 4A, B, C, D). This finding was corroborated by immunohistochemistry, which showed a marked increase in CD68^+^ macrophage infiltration in the spleens of hypoxic mice, particularly in the HM group (Fig. 4E, arrowheads).

At the molecular level, hypoxia robustly upregulated the mRNA expression of the chemokines KC, MCP-1, and MIP-2 in the spleen. Mirroring the cellular infiltration data, the expression levels of all three chemokines were significantly higher in the HM group than in the H group (Fig. 4F, G, H). Collectively, these results demonstrate that hypoxia promotes a strong chemokine-driven inflammatory response in the spleen, which is substantially amplified by MPO deficiency.

### Hypoxia induces a compensatory antioxidant response that is attenuated in MPO-deficient mice

**Fig. 5:**
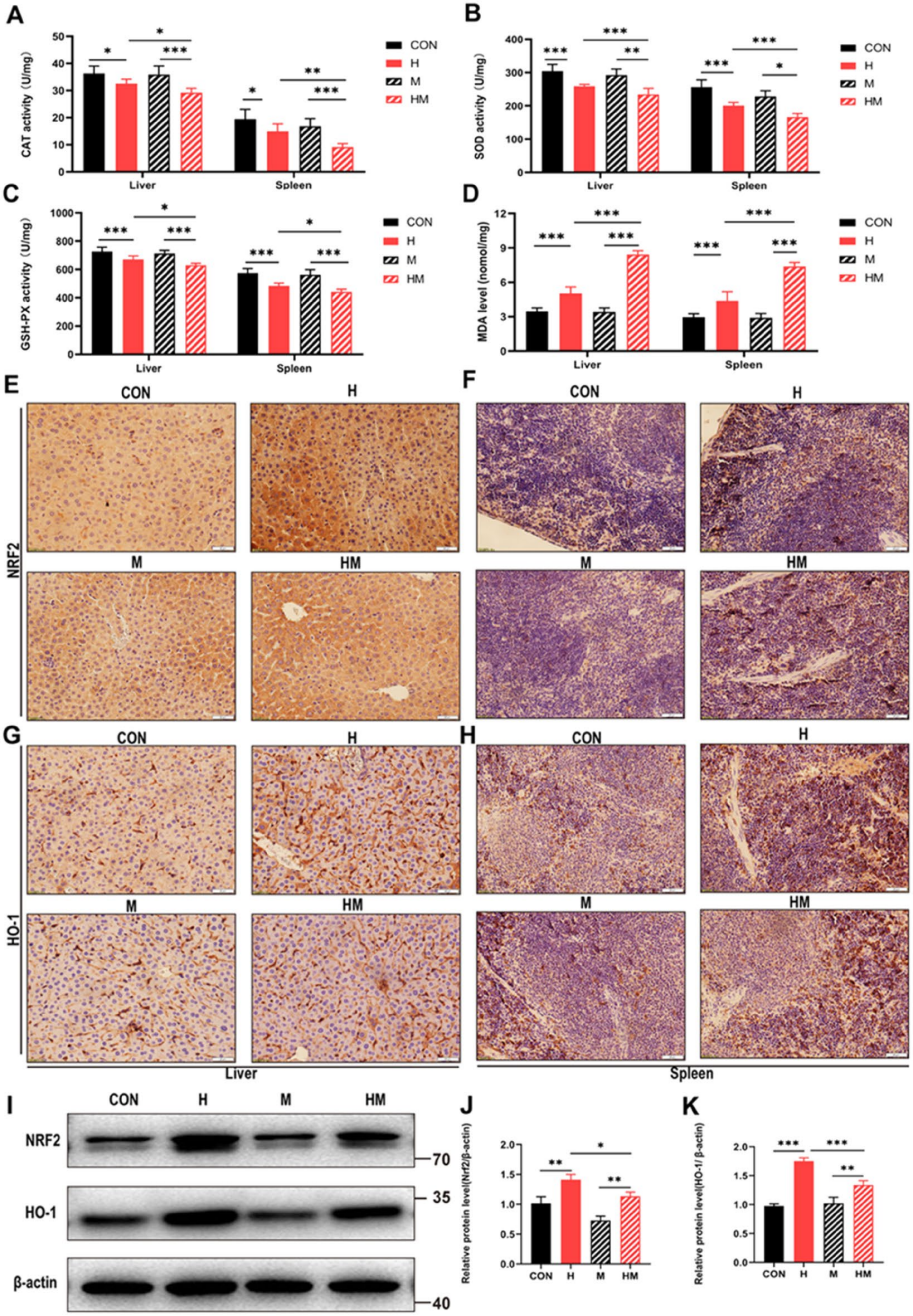
MPO modulates the hypoxic antioxidant response. Analysis of oxidative stress markers in liver and spleen: (**A**) CAT, (**B**) SOD, (**C**) GSH-px activity, and (**C**) MDA level (*n* = 8). Immunohistochemistry for (**E, F**) NRF2 and (**G, H**) HO-1 (×400). (**I**) Representative western blots and quantification of (**J**) NRF2 and (**K**) HO-1 in spleen (*n* = 3). Data are presented as the mean ± SEM and analyzed via one-way ANOVA with Tukey’s multiple comparison test (*p < 0.05; **p < 0.01; ***p < 0.001)

To investigate the role of oxidative stress in hypoxic injury, we evaluated the anti-oxidative status in the liver and spleen. Measurements of key antioxidant enzymes revealed that the activities of CAT, SOD, and GSH-PX were significantly decreased in both tissues following hypoxic exposure in wild-type mice (H group vs. CON group) and, to a lesser extent, in MPO^−^/^−^ mice (HM group vs. M group) (Fig. 5A, B, C). Conversely, the level of MDA, a marker of lipid peroxidation, was significantly increased under hypoxia (Fig. 5D). Notably, the liver exhibited higher basal levels of these oxidative stress indicators compared to the spleen.

We further probed the NRF2/HO-1 signaling pathway, a key regulator of the antioxidant response. Immunohistochemistry demonstrated that hypoxia increased the protein expression of both NRF2 and HO-1 in the liver and spleen. However, this induction was more pronounced in the H group than in the HM group (Fig. 5E, F, G, H). This observation was quantitatively confirmed by western blot analysis of spleen tissues, which showed that the protein levels of NRF2 and HO-1 were significantly up-regulated in the H and HM groups compared to their controls, with a substantially stronger increase in the H group than in the HM group (Fig. 5I, J, K). These findings indicate that hypoxia triggers a robust, MPO-dependent activation of the NRF2/HO-1 pathway as part of the systemic antioxidant defense.

### MPO deficiency exacerbates hypoxia-induced expression of inflammatory proteins in the spleen

**Fig. 6:**
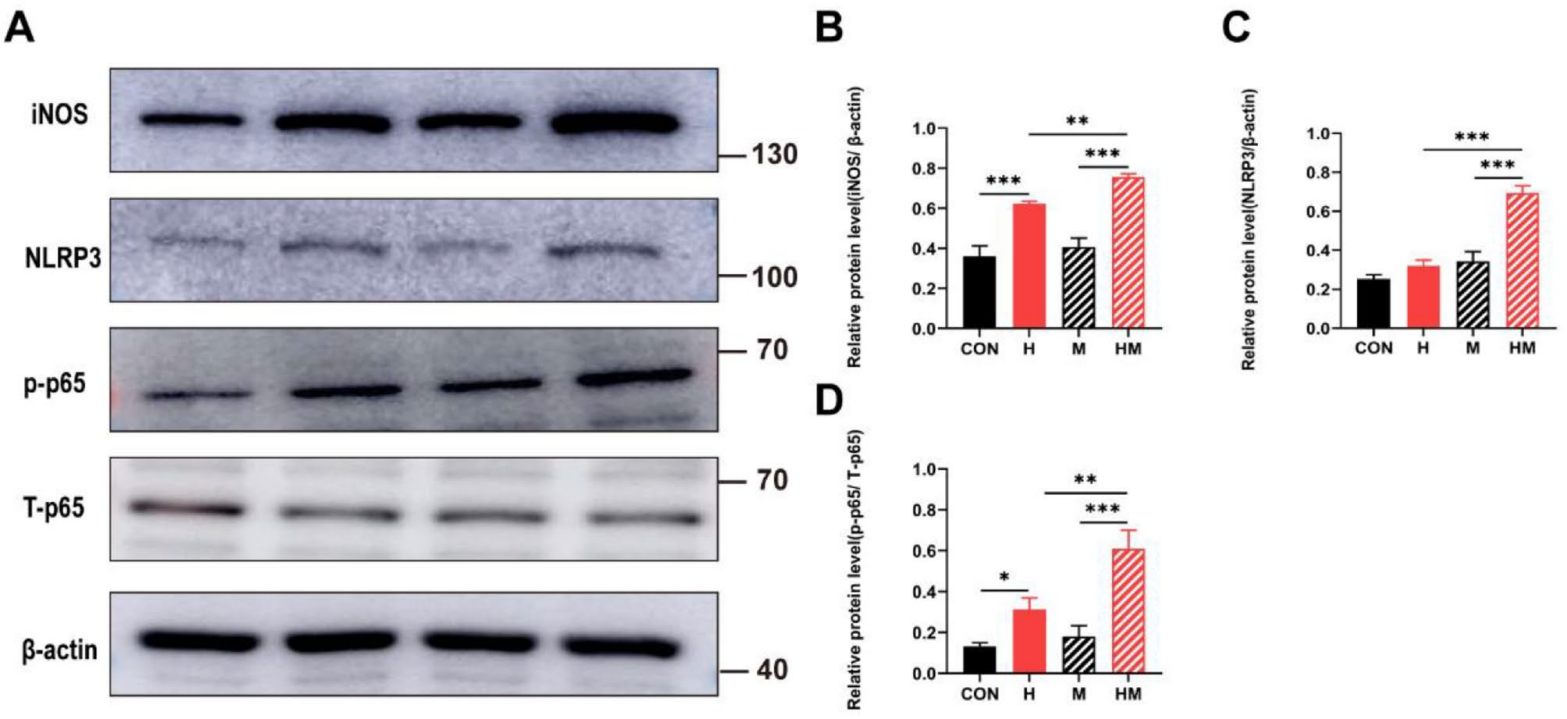
Enhanced inflammatory protein expression in the spleens of hypoxic MPO^−^/^−^ mice. (**A**) Western blot analysis and (**B-D**) quantitative analysis of iNOS, NLRP3, and NF-κB protein expression in spleen tissues (*n* = 3). Data are presented as the mean ± SEM and analyzed via one-way ANOVA with Tukey’s multiple comparison test (*p < 0.05; **p < 0.01; ***p < 0.001)

To further elucidate the pro-inflammatory mechanisms, we analyzed the expression of key inflammatory mediators in the spleen by western blotting. Hypoxic exposure significantly up-regulated the protein levels of inducible nitric oxide synthase (iNOS) and the nuclear factor kappa-B (NF-κB) in the H group compared to the CON group. A similar increasing trend was observed for the NLRP3 inflammasome, although it did not reach statistical significance (Fig. 6A, B, C, D). Strikingly, the absence of MPO markedly potentiated this inflammatory response. The protein levels of iNOS, NF-κB, and NLRP3 were all significantly higher in the HM group than in the H group (Fig. 6A, B, C, D), indicating that MPO deficiency amplifies the activation of multiple pro-inflammatory pathways under hypoxic stress.

## Discussion

Low-pressure hypoxic conditions prevalent at high altitudes can lead to organ damage due to acute hypoxia. The effects of MPO on hepatic and splenic axis inflammation and oxidative stress under hypoxia remain unclear. This study investigated the relationship between MPO and hepatic and splenic axis injury under hypoxia. Our results suggest that short-term exposure to hypoxia at low pressure leads to liver and spleen tissue damage and up-regulation of inflammatory factors, causing tissue oxidative stress response. After MPO knockout, hypoxia will further aggravate liver and spleen injury. Our results also suggest that MPO deficiency further enhances phagocyte recruitment in liver and spleen tissue under hypobaric hypoxia conditions.

It is important to contextualize our unexpected findings within the existing literature. A recent study demonstrated that MPO knockout confers protection against high-altitude pulmonary edema (HAPE) under extreme hypoxic stress (7000 m, 24–48 h) by reducing oxidative stress and preserving alveolar-vascular integrity [38]. In striking contrast, our data obtained under moderate but prolonged hypoxia (5000 m, 72 h) reveal that MPO deficiency significantly worsens oxidative damage and inflammation in the liver and spleen. We propose that these diametrically opposite outcomes highlight the context-dependent, dual role of MPO, critically influenced by the severity and duration of hypoxic insult. In the previous study, extreme altitude (7000 m) likely induced an overwhelming acute oxidative burst that overwhelmed pulmonary defenses. In this scenario, MPO functions as a primary mediator of injury, and its deletion is beneficial. Conversely, at moderate altitude (5000 m), the initial oxidative stress is less severe. We hypothesize that this level of stress may trigger adaptive pathways in peripheral organs over 72 h. While MPO is traditionally viewed as a pro-oxidant enzyme, emerging evidence suggests it may also participate in resolving inflammation or modulating redox-sensitive signaling under certain conditions [39]. In our model, the complete absence of MPO might disrupt this adaptive response, leading to loss of redox homeostasis and paradoxical accumulation of oxidative damage in the liver and spleen during the sub-acute phase. This could involve altered neutrophil function, impaired clearance of damaged cells, or dysregulation of other antioxidant enzymes.

Thus, our findings extend, rather than contradict, the previous work. They suggest that MPO’s pathological role exists on a continuum: from a driver of acute injury under extreme stress to a potential modulator of adaptation under moderate, sub-acute stress. This study underscores that therapeutic targeting of MPO may have differential, even opposing, effects depending on hypoxia severity and the organ system involved.

The characteristics of high-altitude environments are more sensitive to the effect of the hematopoietic system, and the change in blood routine is one of the earliest sensitive indicators of altitude disease [40]. Blood routine can be divided into three systems, namely, the red blood cell system, the white blood cell system, and the platelet system, which is of great significance for the overall evaluation of the severity of hypoxia injury at altitude [41]. Our data showed that blood routine RBC levels in WT and MPO^-/-^ mice were significantly elevated 72 hours after hypoxic exposure, and the results were consistent with Wang’s [42] study, indicating that exposure to hypoxia can cause stress erythropoiesis. The data also showed a significant increase in the spleen index, suggesting stress erythropoiesis in the spleen to compensate for reduced oxygen supply during hypoxia, leading to splenomegaly. The level of spleen index can reflect the degree of lymphocyte proliferation and can roughly estimate the strength of immune function. Three main factors can cause splenomegaly: increased splenic function, infiltration, or congestion [43]. Therefore, splenomegaly is caused by increased splenic function during stress erythropoiesis. After the body rushes into altitude hypoxia, the stress response will activate many WBCs, leading to pathological changes such as lipid peroxidation and a large number of white blood cells exudation, resulting in inflammation. These results indicate that the low oxygen environment at high altitudes can increase WBC, Neu, and RBC.

The results showed that the expression of inflammatory factors TNF-α, IL-17A, and IL⁃1β in liver and spleen tissue increased significantly after hypoxia, indicating that hypoxia at high altitudes can induce the increase of the expression of various pro-inflammatory cytokines. Some animal and human studies in high-altitude areas have found elevated levels of inflammatory factors [44]. TNF-α, IL-17A, and IL⁃1β are important pro-inflammatory factors, and their activity levels can indicate inflammatory damage in the body [10, 45].

Pro-inflammatory cytokines, such as IL-1β, trigger the “typical” pathway of NF-κβ activation. In vitro experiments have shown that increased IL-1β levels can enhance the proliferation of stressed red blood cell progenitors [16, 17]. In our study, the expression of inflammatory factors (IL-1β, TNF) in spleen tissues was significantly increased under hypoxia, accompanied by an upregulation of red blood cell count, and the expression of NF-κB protein was increased in WB results. Therefore, consideration of the NF-κB signaling pathway may be a key pathway to regulating immune response.

We noted that when the mice were exposed to hypobaric hypoxia, antioxidant defense factors in the liver and spleen, such as MDA, were significantly increased. In contrast, GSH-Px, SOD, and CAT levels were decreased. This result indicated that the antioxidant capacity was weakened, and liver and spleen oxidation were aggravated after hypoxia. And the liver is more sensitive to oxygen supply. Previous studies have shown that the production and activity of antioxidant defense factors were inhibited by inflammatory factors [46], accompanied by a significant increase in inflammatory factors. Therefore, the results of this study suggest that exposure to high altitudes can disrupt the antioxidant system, leading to oxidative damage and inflammatory responses.

Through experiments, we observed that the expression level of the NRF2/HO-1 pathway in the spleen of mice in the H group and HM group was significantly up-regulated compared with that in the CON group and M group, respectively. This upregulation typically indicates an enhanced antioxidant capacity. However, the measured oxidative stress indicators showed a contradictory pattern: MDA was significantly increased, while the activities of SOD, CAT, and GSH-PX were significantly decreased under hypoxia. These results suggest that the actual antioxidant capacity was weakened and oxidative damage in the spleen was aggravated.

This observed dissociation between NRF2/HO-1 pathway activation and reduced antioxidant enzyme activities differs from the consistent correlation reported in some previous studies [47, 48]. While it is well-established that the NRF2/HO-1 signaling pathway is crucial for protecting cells against oxidative stress [49], our findings reveal a more complex regulatory scenario. To explain this apparent contradiction, we propose the following interconnected mechanisms: (1) Compensatory upregulation with impaired downstream function. The activation of the NRF2/HO-1 pathway may represent a compensatory increase in response to hypoxic stress, similar to the concept of “insulin resistance” in obesity [50]. In this state, although the upstream signaling is activated, the actual execution of antioxidant function (e.g., by SOD, CAT, GSH-PX) is impaired, preventing effective protection against oxidative damage. (2) Post-translational modifications or oxidative inactivation of antioxidant enzymes. Despite increased NRF2-mediated transcription, the activities of SOD, CAT, and GSH-PX may be directly suppressed by post-translational modifications or oxidative damage caused by excessive ROS. For example, high levels of ROS can oxidize critical cysteine residues or alter the protein structure of these enzymes, leading to their inactivation and subsequent loss of function. (3) Exhaustion of compensatory capacity under chronic stress. The 72-hour hypoxic exposure in our model may represent a prolonged stress that overwhelms the compensatory capacity of the NRF2/HO-1 pathway. While this pathway is rapidly activated, its protective effects may be insufficient to counteract the sustained and cumulative oxidative damage, leading to a net decrease in antioxidant enzyme activity and increased lipid peroxidation (MDA). This interpretation is consistent with the temporal dynamics reported in human studies. For instance, in subjects rapidly exposed to a 3830 m hypoxic environment, peak expression levels of HIF-2α and NRF2 were observed at 72 hours [51]. Conversely, volunteers exposed to acute hypobaric hypoxia (4220 m, 36 h) showed a pro-oxidation/antioxidant imbalance with increased MDA and decreased GPX activity as early as 24 hours [2]. Similarly, Tang [52] found that plasma SOD activity decreased after 24 hours at 3500 m altitude. These time-course observations suggest that NRF2 activation and antioxidant enzyme function are not always synchronized, and that functional decline can occur despite transcriptional upregulation, particularly under sustained hypoxic conditions.

An important question raised by our findings is which specific splenic cell populations are responsible for the observed Nrf2/HO-1 pathway activation. While our measurements in whole spleen homogenates reflect the cumulative response of multiple cell types, they do not allow definitive identification of the responsible populations. Based on the existing literature, we hypothesize that macrophages and lymphocytes are the most likely contributors. Macrophages are known to upregulate Nrf2/HO-1 signaling in response to oxidative stress, and lymphocytes exhibit Nrf2-dependent regulation of differentiation and function. Other cell types, including dendritic cells and endothelial cells, may also participate. Future studies using immunohistochemical co-localization or flow cytometry-based intracellular staining with cell-specific markers will be necessary to definitively resolve this question.

In this study, it was found that hypoxia-induced increased expression of chemokines in phagocytes (liver and spleen), which promoted recruitment of Ly6G^+^ neutrophils and F4/80^+^ macrophages, indicating that hypoxia could induce recruitment of phagocytes, lead to respiratory burst, produce a large number of reactive oxygen species, and then impair cell function. Moreover, MPO deficiency led to a marked increase in the recruitment of polymorphonuclear neutrophils (PMN) in the liver and spleen tissues in hypoxia. Research confirms that the MPO level is routinely measured as an indirect enzymatic method to quantify the level of neutrophil infiltration in tissue and reflect neutrophil activation [53]. Furthermore, MPO can regulate the interaction between neutrophils and immune cells and coordinate the immune response [54]. In this study, despite the absence of MPO, the recruitment of phagocytes and neutrophils in liver and spleen tissue was promoted, suggesting that other mechanisms regulate PMN function.

These findings prompt a critical inquiry into which specific MPO-expressing cell population drives the hypotensive hypoxemia observed in hypoxic MPO^-/-^ mice (HM). MPO is predominantly expressed in neutrophils, with lower levels reported in monocytes and in tissue-resident macrophages under baseline conditions [55, 56]. Given the acute inflammatory context of our 72-hour hypoxic exposure and the established role of neutrophils as early responders to hypoxic stress [57], we propose that neutrophil-derived MPO is the most likely driver of the HM phenotype. This hypothesis is supported by the temporal dynamics of neutrophil recruitment, which peaks within 24–72 hours of inflammatory stimuli, coinciding with the onset of HM in our model. Furthermore, the high concentration of MPO in neutrophil azurophilic granules makes neutrophils the most potent source of MPO-mediated oxidative damage during acute inflammation. Nevertheless, we cannot exclude a partial contribution from MPO expressed in recruited monocytes, which infiltrate tissues and differentiate into macrophages over a similar time course. Monocyte-derived macrophages may contribute to the sustained inflammatory response and tissue damage observed at 72 hours. Elucidating the precise cellular source of pathogenic MPO will require more sophisticated genetic approaches, such as cell-type-specific MPO knockout models (e.g., targeting neutrophils specifically) or adoptive transfer experiments. These represent important avenues for future investigation to better understand the cellular mechanisms underlying HM in MPO deficiency.

Low pressure and hypoxia increase H_2_O_2_ content and NADPH oxidase expression [58], and an explosion of oxidation occurs in neutrophils and macrophages [59, 60]. Neutrophils and monocytes express MPO. A study on human neutrophils showed a negative correlation between MPO activity levels and extracellular H_2_O_2_ release after *Salmonella* stimulation [61]. Studies have shown that MPO-deficient neutrophils accumulate large amounts of H_2_O_2_, which leaks out of the phagosome. Therefore, it is considered that in this study, the level of H_2_O_2_ is also higher in MPO-KO mice during hypoxia, and the oxidative stress reaction is aggravated, further aggravating the damage to the host organ tissues.

Our study observed that the expression level of iNOS protein in the spleen of WT mice and MPO knockout mice was higher under hypoxic conditions. Hypoxia can directly affect the vascular tension of systemic resistance vessels, resulting in decreased expression and function of endothelial nitric oxide synthase (eNOS) [62]. MPO acts as a key oxidase that impairs the biological activity of nitric oxide (NO) [63] and promotes endothelial dysfunction. Combined with the above, it can be seen that hypoxia and MPO have similar effects. If the two factors are superimposed, the effect doubles.

HIF1α is a key transcription factor controlling innate immune function [64]. M1-type macrophages produce pro-inflammatory cytokines, such as TNF, IL-1β, IL-6, ROS, and NO [65]. If macrophages are over-activated, the excessive secretion of inflammatory cytokines may damage tissues. Excessive accumulation and activation of neutrophils, along with the release of various proteolytic enzymes, also lead to tissue damage. Therefore, the innate immune system of the body is a double-edged sword. Whether it is too low or too high, it may cause damage to the body.

Several limitations of this study should be acknowledged. First, regarding experimental design limitations. Although our comparative analysis of MPO-KO and WT mice provides strong genetic evidence for the role of MPO in hypoxia-induced inflammation, we did not perform rescue experiments to restore MPO expression in KO mice. Such experiments represent the gold standard for confirming gene function, and their absence limits our ability to draw definitive causal conclusions. Future studies should employ viral-mediated gene delivery (e.g., AAV vectors) to reconstitute MPO expression in the liver and spleen of KO mice, or adoptive transfer of MPO-expressing neutrophils to test cell-type-specific effects. However, given the technical complexity and extended timeline, such experiments were beyond the scope of the current study. Nevertheless, the multi-level evidence presented—including direct MPO activity measurements, consistent KO versus WT comparisons across multiple endpoints, and mechanistic alignment with MPO’s known biology—provides robust support for our conclusions. Rescue experiments are now highlighted as a priority for future research.

Second, regarding limitations in immune assessment and functional characterization. (1) Incomplete peripheral immune evaluation: While we comprehensively evaluated immune responses in the liver and spleen, we did not assess peripheral blood lymphocyte populations. Future studies should include such analyses to elucidate systemic immune alterations under hypoxic exposure. (2) Lack of whole-organism physiological assessments: Our study focused on molecular, histological, and biochemical endpoints in the liver and spleen, but did not perform functional physiological assessments (e.g., lung function tests, arterial blood gas analysis, or cardiovascular parameters). Such measurements are essential to determine whether tissue-level changes translate to whole-organism physiological consequences. (3) Absence of organ function biomarkers: Although histological analysis revealed structural changes in the liver, we did not assess liver function through serum biomarkers (e.g., ALT, AST). Future studies should include these measurements to establish the functional significance of the observed hepatic pathology. (4) Insufficient functional immune characterization: While flow cytometry identified changes in myeloid cell populations, we did not perform functional immune assays (e.g., phagocytosis, bacterial killing, or response to ex vivo stimulation). Such experiments would elucidate whether MPO deficiency alters immune cell effector function beyond changes in cell numbers. These limitations represent important avenues for future investigation.

Under hypoxic conditions, the liver and spleen showed consistent manifestations in terms of inflammation, oxidative stress, and phagocyte recruitment, suggesting that there is a close synergistic effect between the two organs in these aspects. This synergy may be achieved through shared signaling pathways, cytokine networks, or inter-organ communication. However, this study did not explore the specific mechanism of this synergy. This part will be analyzed in depth as a focus of the following research.

## Conclusions

In conclusion, our findings delineate a critical and dual-layered role for MPO in the pathophysiology of hypoxic organ injury. Beyond its established pro-inflammatory function, MPO serves as an essential modulator that constrains the overall inflammatory response and facilitates the activation of the compensatory NRF2/HO-1 antioxidant pathway. The genetic ablation of MPO disrupts this delicate balance, leading to a hyper-inflammatory state and heightened oxidative damage in the liver and spleen. Therefore, therapeutic strategies targeting MPO in hypoxic conditions must be carefully considered, as complete inhibition may paradoxically worsen tissue injury by disinabling these vital regulatory functions.

## Data Availability

The datasets used and/or analyzed during the current study are available from the corresponding author on reasonable request.
